# Numerical Simulations of Magnetohydrodynamics Natural Convection and Entropy Production in a Porous Annulus Bounded by Wavy Cylinder and Koch Snowflake Loaded with Cu–Water Nanofluid

**DOI:** 10.3390/mi13020182

**Published:** 2022-01-26

**Authors:** Abed Mourad, Aissa Abderrahmane, Obai Younis, Riadh Marzouki, Anas Alazzam

**Affiliations:** 1 Laboratoire de Physique Quantique de la Matière et Modélisation Mathématique (LPQ3M), University Mustapha Stambouli of Mascara, Mascara 29000, Algeria; mourad.abed@univ-mascara.dz; 2 Department of Mechanical Engineering, College of Engineering at Wadi Addwaser, Prince Sattam Bin Abdulaziz University, Al-Kharj 16278, Saudi Arabia; oubeytaha@hotmail.com; 3 Department of Mechanical Engineering, Faculty of Engineering, University of Khartoum, Khartoum 11111, Sudan; 4 Chemistry Department, College of Science, King Khalid University, Abha 61413, Saudi Arabia; rmarzouki@kku.edu.sa; 5 Department of Mechanical Engineering, Khalifa University, Abu Dhabi P.O. Box 127788, United Arab Emirates

**Keywords:** magnetohydrodynamic, natural convection, Koch snowflake, hybrid nanofluid, porous media

## Abstract

The current paper presents a numerical study of the magnetohydrodynamics natural convection and entropy production of Cu–water nanofluid contained in a porous annulus between a heated Koch snowflake and wavy cylinder with lower temperature with respect to the Koch snowflake. The numerical algorithm is based on the Galerkin Finite Element Method. The impacts of Rayleigh number (*Ra* = 10^3^, 10^4^, 10^5^, and 10^6^), Hartman number (*Ha* = 0, 25, 50, and 100), Darcy number (*Da* = 10^−2^, 10^−3^, 10^−4^, and 10^−5^), nanoparticle volumetric fraction (*φ* = 2%, 3%, 4%, and 5%), and the undulations number of the outer wavy cylinder (three cases) on the distributions of isotherms, streamlines, mean Nusselt number (*Nu_avg_*), as well as on total entropy production and Bejan number are thoroughly examined. The computational outcomes disclose that dispersing more Cu nanoparticles in the base fluid and creating a flow with higher intensity inside the annulus by raising the Rayleigh number bring about a boosted natural convective flow in the cavity, which improves the heat transmission rate. In addition, it can be noted that owing to the peculiar form of the heated Koch snowflake, nanofluid gets trapped between the angled parts, resulting in uneven temperature profiles with higher values in these places.

## 1. Introduction

Convective heat transfer via a mix of materials has become an important technique in a range of industrial and residential applications, including building heating and cooling, thermal management of electronic components, systems of heat exchange, and solar energy. Several researchers have examined the characteristics of fluid flow and heat transmission in numerous technical applications [1,2,3,4]. In the last decade, high-conductivity nanoparticles have been presented as a means of enhancing heat transmission [5,6,7,8]. The dissolving of nanoparticles in the base fluid produces a fluid known as a nanofluid. An annular channel is one of the most significant concepts in thermal energy systems. They are used in a variety of heat and mass transfer technologies. Numerous researchers have examined the impact of geometric characteristics of the annulus, the thermophysical properties of the working fluid, such as nanofluids, and internal or external factors on free convective flow and heat transfer rate in numerous computational and experimental studies [9,10,11,12,13,14].

Al-Rashed et al. [15] examined 3D convection heat transfer in a parallelogram open enclosure loaded with nanofluid and a partially heated square on the bottom side. The collected results demonstrated that the tilting angle and volume percentage of nanoparticles have an effect on the flow structure and promote heat transmission. Mirzaie et al. [16] numerically explored the convection heat transfer coefficient of a Cu–water nanofluid loaded within an annulus with pair emitters and inner revolving circular cylinder. Boulahia et al. [17] explored the irreversibility of 3D and 2D MHD free convective flow inside a cubical-shaped container filled with hybrid water nanofluid. According to their findings, altering the Rayleigh number, Hartmann number, and magnetic field orientation considerably improves heat transport and entropy generation. Additionally, it is discovered that raising the volume percentage of hybrid nanoparticles may be tuned to achieve a significant increase in heat transmission rate. Aljabair et al. [18] presented numerical simulations of free convective heat transmission in symmetrical and asymmetrical corrugated annuli loaded with an H_2_O-Al_2_O_3_ nanofluid. Miles et al. [19] researched laminar natural convective flow and the production of entropy in a vertical porous annulus saturated with nanofluids. They discovered that the introduction of nanoparticles enhances heat transport and boosts overall entropy generation. Asiaei et al. [20] examined the heat transmission and entropy formation of a copper–water nanofluid inside a two-sided lid-driven cavity containing a heat source and a multi-layered porous media. The results suggest that employing the multi-layered porous material limits the extension of flow vortices near the moving walls, hence increasing heat transmission by up to 17%. Seyyedi et al. [21] explored the formation of entropy in a semi-annulus cavity loaded with a copper–water nanofluid with various nanoparticles shapes. The findings indicate that when the Rayleigh number and the nanoparticles concentration grow, the Nusselt number and entropy production rise as well. Bouzerzour et al. [22] examined free convective flow in Cu–water nanofluid and a partly heated annulus formed by the intersection of two elliptical cylinders by means of numerical simulations. They discovered that mounting the heaters on the left and right sides of the inner cylinder wall results in improved heat transmission. Yu-Peng Hu et al. [23] researched the free convective flow of a water-based nanofluid in an annulus at its density maximum. Motlagh et al. [24] demonstrated two-phase mathematical modeling of nanofluid natural convection in a tilted porous semi-annulus container. They observed that elevating the tilting angle of the container lowers the heat transfer rate for large porous Rayleigh numbers. Furthermore, whenever the porosity number is increased, the Nusselt number increased.

Natural convection occurs in various industrial processes, including cooling fusion reactors and crystal formation in liquid. Numerous research studies have been published in the literature to describe natural convection and heat transmission of nanofluids inside a container subject to magnetic fields. Researchers have discovered that the magnetic field’s direction and strength are two critical factors affecting nanofluid’s thermal performance and flow patterns. Hatami et al. researched the impact of a fluctuating magnetic field on the temperature distribution inside a half-annulus enclosure loaded with Fe_3_O_4_–water nanofluid while the heat flux was constant. Yan Cao et al. [25] explored the natural convection of a water–Al_2_O_3_ nanofluid within a cold semi-annulus chamber equipped with a heated block positioned at the bottom surface. At lower values of Rayleigh numbers, raising the volumetric fraction boosts the Nusselt number. Additionally, for large Rayleigh numbers, the Nusselt number is in inverse proportion to the Hartmann number. Abderrahmane et al. explored the non-Newtonian nanofluid MHD free convective flow within a halved annular container, in which the linear walls of the container were subjected to a uniform heat flux. Their results suggest that the cavity’s inclination angle and Hartmann number may be used as efficient control factors. Dogonchi et al. [26] explored the impact of thermal radiation and free convection on the thermodynamics of nanofluid heat transmission in an annulus created by a wavy circular cylinder within a rhombus enclosure in being subjected to a uniform magnetic field. In another study [27], they considered a porous annulus developed between an elliptical cylinder and square cavity. Hadidi et al. [28] researched the convective heat transfer of a non-Newtonian nanofluid flow loaded into an internally finned annulus and exposed to external magnetic field. Their results suggested that when nanoparticles are mixed with the base fluid, the augmentation in total heat transfer rate is more prominent than before, especially at lower Rayleigh number values. In addition, the distinction between the equivalent thermal conductivities for varying volume fractions decline as the Hartmann number increases. In the existence of a magnetic field, Seyyedi et al. [29] investigated heat transport and entropy formation in a semi-annulus porous enclosure loaded with Cu–water nanofluid. The findings indicate that when the Rayleigh number and the nanoparticle volumetric fraction grow, the Nusselt number and entropy production rise as well. Sheikholeslami et al. [30] investigated magnetohydrodynamic flow in an inclined enclosure loaded with nanofluid. The findings indicate that the applied magnetic field slowed the velocity field, resulting in a reduction in convection and Nusselt number. Gupta et al. [31] analyzed the impact of a magnetic field, magnetic field tilting angle, and the number of undulations on the free convective heat transmission of a hybrid nanofluid inside a wavy annulus with a centered circular heater and separate coolers along the wavy walls. Tayebi et al. [32] reconsidered the free convective flow mechanism in a homocentric circular annulus formed by a heat-generative conducting interior cylinder and isothermal outer cylinder at lower temperature loaded with a carbon nanotubes–water nanofluid.

As demonstrated by the prior research review, annuli enclosures play a critical role in thermal convection heat transfer in a variety of thermal energy technologies. Despite this, scientists have not previously considered the investigation of free convective flow and heat transfer inside a homocentric annulus porous gap established between an inner heat-generative conducting Koch snowflake cylinder and a cold external corrugated cylinder loaded with Cu–water nanofluid and exposed to an external magnetic field. As the Koch snowflake shape increases the surface area and hence the heat transfer rate, there were some recommendations for it to be used in heat exchangers [33,34].

The main question we are exploring here is the direct influence of the presence of complex geometry as a heat-generating element on fluid motion and heat exchange. As a consequence, we feel that this study is distinctive and beneficial. The numerical simulations were established by using the Finite Element Method (FEM). The visualization results for flow and heat transfer are presented as isotropic lines, isotherms, and streamlines contours for a variety of Hartmann numbers, Darcy numbers, nanoparticles concentrations, and geometrical factors. Additionally, the average Nusselt values for the heated walls and the Bejan value are computed and analyzed numerically.

## 2. Physical Model Description

**Figure 1:**
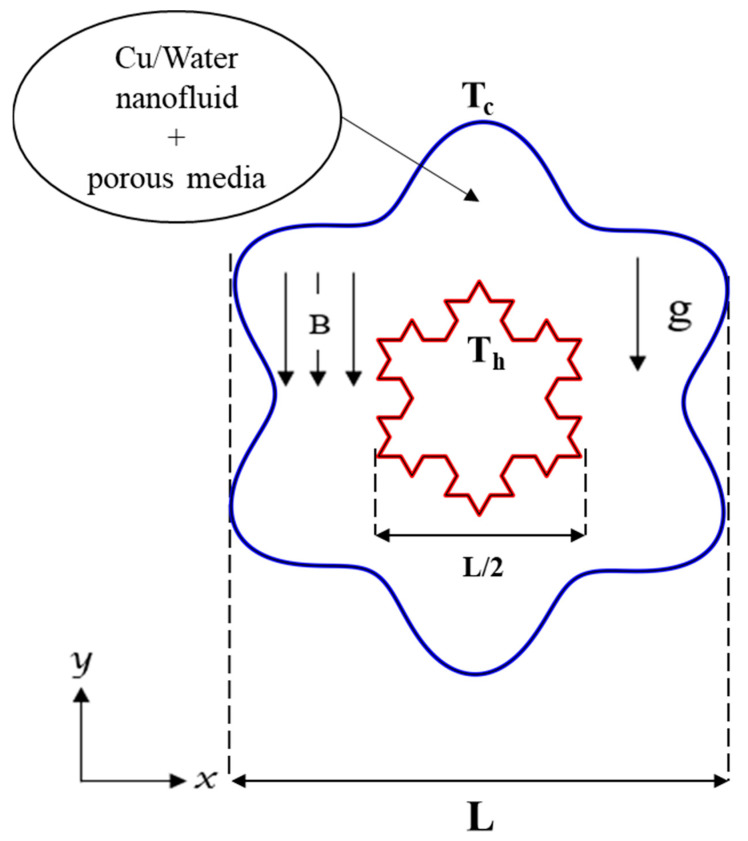
The schematic representation of the current study.

For the present problem, two-dimensional, unsteady, laminar natural convection flow in an annulus bordered by a Koch snowflake and a wavy cylinder is analyzed. The computational and physical domains and the involved boundary conditions are presented in Figure 1. The figure represents an outside cold wavy cylinder (*T_c_*) and an interior hot Koch snowflake (*T_h_*). The porous annulus space with outer diameter *L* and inner diameter *L*/2 is saturated with a Newtonian nanofluid. Cu–water nanofluid was utilized in this research, and their respective thermophysical properties are listed in Table 1. Thermal equilibrium is considered between nanoparticles representing the solid phase and water representing the liquid phase, and both water and nanoparticles have the same motion (i.e., same magnitude and direction of flow).

With regard to the transport procedures, the following assumptions are considered:

- Thermal equilibrium is postulated between the base fluid and the nanoparticles. Moreover, there is no slip effect between the copper nanoparticles and water.
- The flow is considered to be incompressible, continuous, 2D, and laminar.
- Newtonian nanofluid.
- Except for the density in the body force component, which is approximated using the Boussinesq approximation, the fluid parameters are assumed to remain constant throughout the simulation.
- Heat transmission by radiation is disregarded.
- The effects of Joule heating, viscous dissipation, and displacement currents are deemed insignificant.

## 3. Mathematical Modeling

### 3.1. Governing Equations

Brinkman’s model [36] is used to mathematically reflect permeable media. On the basis of the above-mentioned assumptions, the following dimensional form may be used to define the governing equations for mass, momentum, and energy of the problem under study:

Continuity:

(1) $$\frac{\partial \left(u\right)}{\partial x}+\frac{\partial \left(v\right)}{\partial y}=0$$

*X*-direction component of the momentum:

(2) $$u\frac{\partial u}{\partial x}+v\frac{\partial u}{\partial y}=-\frac{1}{\left(\rho \right)}\frac{\partial p}{\partial x}+\frac{\left(\mu \right)}{\left(\rho \right)}\left(\frac{\partial^{2}u}{\partial x^{2}}+\frac{\partial^{2}u}{\partial y^{2}}\right)-\frac{{\left(\mu \right)}_{}}{\left(\rho \right)K}u$$

*Y*-direction component of the momentum:

(3) $$u\frac{\partial v}{\partial x}+v\frac{\partial v}{\partial y}=-\frac{1}{\left(\rho \right)}\frac{\partial p}{\partial y}+\frac{\left(\mu \right)}{\left(\rho \right)}\left(\frac{\partial^{2}v}{\partial x^{2}}+\frac{\partial^{2}v}{\partial y^{2}}\right)+\left(\beta \right)g\left(T-T_{c}\right)-\frac{\left(\mu \right)}{\left(\rho \right)K}v-\frac{\sigma B_{0}{}^{2}}{\left(\rho \right)}v.$$

Energy:

(4) $$u\frac{\partial T}{\partial x}+v\frac{\partial T}{\partial y}=\alpha \left[\left(\frac{\partial^{2}T}{\partial x^{2}}+\frac{\partial^{2}T}{\partial y^{2}}\right)\right]$$

To rebuild the equations in a dimensionless form, the following requirements have been considered:

Dimensionless scales

(5) $$X,Y=\frac{x,y}{L},U,V=\frac{\left(u,v\right)L}{\alpha_{fl}},\theta =\frac{T-T_{c}}{T_{h}-T_{c}}$$

Dimensionless numbers:

(6) $$P=\frac{pL^{2}}{\rho \alpha_{fl}^{2}}$$

(7) $$Ra=\frac{g\beta_{fl}(T_{h}-T_{c})L^{3}}{\alpha_{fl}v_{fl}}\left(Rayleigh\;number\right)$$

(8) $$Da=\frac{\lambda }{L^{2}}\left(Darcy\;number\right)$$

(9) $$Pr=\frac{v_{fl}}{\alpha_{fl}}(Prandtl\;number)$$

The following correlations describe the properties of nanofluid and are founded on classical models found in the literature [37]:

(10) $$\rho_{nf}=(1-\phi )\rho_{fl}+\phi \rho_{s}$$

(11) $$\sigma_{nf}=(1-\phi )\sigma_{fl}+\phi \sigma_{s}$$

(12) $${\left(\rho c_{p}\right)}_{nf}=(1-\phi ){\left(\rho c_{p}\right)}_{fl}+\phi {\left(\rho c_{p}\right)}_{s}$$

(13) $${\left(\rho \beta \right)}_{n}{}_{f}=\left(1-\phi \right)\rho_{fl}+\phi {\left(\rho \beta \right)}_{s}$$

(14) $$\alpha_{nf}=\frac{K_{nf}}{{\left(\rho c_{p}\right)}_{nf}}$$

where ϕ represents the volumetric fraction of the solid nanoparticles, ρ represents the density, thermal conductivity is denoted *K*, σ represents the electrical conductivity, *c_p_* denotes the specific heat, the fluid thermal expansion coefficient is denoted by *α*, and thermal diffusivity is denoted by β.

Brinkman’s model [30] and Maxwell’s model [38] are employed in order to identify both nanofluid’s effective dynamic viscosity and thermal conductivity, respectively. The resultant relations read:

(15) $$\frac{K_{nf}}{K_{fl}}=\frac{K_{s}+2K_{fl}-2\phi (K_{fl}-K_{s})}{K_{s}+2K_{fl}+2\phi (K_{fl}-K_{s})}$$

(16) $$\mu_{nf}=\frac{\mu_{fl}}{{\left(1-\phi \right)}^{0.25}}$$

As illustrated below, Equations (1)–(4) are translated into a dimensionless form:

Continuity:

(17) $$\frac{\partial \left(U\right)}{\partial X}+\frac{\partial \left(V\right)}{\partial Y}=0.$$

Momentum in *X* direction:

(18) $$U\frac{\partial U}{\partial X}+V\frac{\partial U}{\partial Y}=-\frac{\partial P}{\partial X}+\frac{{\left(\rho \right)}_{bf}}{\left(\rho \right){\left(1-\phi \right)}^{2.5}}Pr\left(\frac{\partial^{2}U}{\partial X^{2}}+\frac{\partial^{2}U}{\partial Y^{2}}\right)-\frac{{\left(\rho \right)}_{bf}}{\left(\rho \right){\left(1-\phi \right)}^{2.5}}\frac{Pr}{Da}U.$$

Momentum in *Y* direction:

(19) $$U\frac{\partial V}{\partial X}+V\frac{\partial V}{\partial Y}=-\frac{\partial P}{\partial Y}+\frac{{\left(\rho \right)}_{bf}}{\left(\rho \right){\left(1-\phi \right)}^{2.5}}Pr\left(\frac{\partial^{2}V}{\partial X^{2}}+\frac{\partial^{2}V}{\partial Y^{2}}\right)+\frac{\left(\rho \beta \right)}{\rho \beta_{bf}}RaPr\theta -\frac{{\left(\rho \right)}_{bf}}{\left(\rho \right){\left(1-\phi \right)}^{2.5}}\frac{Pr}{Da{\frac{{\left(\rho \right)}_{bf}}{\left(\rho \right){\left(1-\phi \right)}^{2.5}}}^{2}PrV}.$$

Energy:

(20) $$U\frac{\partial \theta }{\partial X}+V\frac{\partial \theta }{\partial Y}=\frac{\alpha }{\alpha_{bf}}\left[\left(\frac{\partial^{2}\theta }{\partial X^{2}}+\frac{\partial^{2}\theta }{\partial Y^{2}}\right)\right].$$

### 3.2. Boundary Conditions

The boundary conditions associated with Equations (17)–(20) are:

Wavy circular outer cylinder perimeter:

θ=0,U=0,V=0.

On the Koch snowflake perimeter:

θ=1,U=0,V=0.

The flow regime through the enclosure was illustrated by streamlines generated by utilizing the following mathematical model

(21) $$\frac{\partial^{2}\psi }{\partial X^{2}}+\frac{\partial^{2}\psi }{\partial Y^{2}}=\frac{\partial U}{\partial Y}-\frac{\partial V}{\partial X}.$$

The heat transfer can be evaluated by means of local and average Nusselt number (*Nu_loc_* and *Nu_avg_*) near the Koch snowflake hot surface; the flowing equations are utilized to calculate them:

(22) $$Nu_{loc}=-\frac{k_{eff}}{k_{fl}}\frac{\partial \theta_{po}}{\partial n}$$

(23) $$Nu_{avg}=\frac{\int_{0}^{S}Nu_{loc}dS}{S}.$$

Due to the coupled magnetohydrodynamic processes, the physical system under consideration incurs irreversibilities. Thus, the entropy created by this process is formed by three components: irreversibility due to local temperature gradients, the influence of viscous dissipation, and the presence of a magnetic field.

According to linear transport theory’s local thermodynamic equilibrium, the rate of local entropy creation inside the enclosure may be stated in dimensional form as:

(24) $$S_{gen}=\frac{K_{nf}}{T_{o}^{2}}\left[{\left(\frac{\partial T}{\partial x}\right)}^{2}+{\left(\frac{\partial T}{\partial y}\right)}^{2}\right]+\frac{\mu_{nf}}{T_{o}}\left[2\left\{{\left(\frac{\partial u}{\partial x}\right)}^{2}+{\left(\frac{\partial v}{\partial y}\right)}^{2}\right\}+{\left(\frac{\partial u}{\partial y}+\frac{\partial v}{\partial x}\right)}^{2}\right]+\frac{\sigma_{nf}B_{o}^{2}}{T_{o}^{2}}v^{2}.$$

The dimensionless formulation of the local entropy generation rate may be stated as:

(25) $$\begin{array}{c} S_{\theta }=\frac{K_{nf}}{K_{f}}\left[{\left(\frac{\partial \theta }{\partial x}\right)}^{2}+{\left(\frac{\partial \theta }{\partial Y}\right)}^{2}\right] \end{array}$$

(26) $$\begin{array}{c} S_{\psi }=\xi \frac{\mu_{nf}}{\mu_{f}}\left[2\left\{{\left(\frac{\partial U}{\partial X}\right)}^{2}+{\left(\frac{\partial V}{\partial Y}\right)}^{2}\right\}+{\left(\frac{\partial U}{\partial Y}+\frac{\partial V}{\partial X}\right)}^{2}\right] \end{array}$$

(27) $$\begin{array}{c} S_{mf}=\xi \frac{\rho_{\mathrm{pf}}}{\rho_{t}}Ha^{2}V^{2} \end{array}$$

(28) $$S=S_{\theta }+S_{\psi }+S_{mf}$$

where *S_θ_*, *S_ψ_*, and *S_mf_* are used to represent the dimensionless entropy generated by heat transfer, fluid friction, and magnetic field, respectively, and *ξ* is used to represent the irreversibility distribution ratio, which may be described as:

(29) $$\xi =\frac{\mu_{f}T_{o}}{K_{f}}{\left(\frac{\alpha_{f}}{L\Delta T}\right)}^{2}$$

In Equations (24) and (25), the value of *ξ* is constant and equal to 100. The production of global entropy may be computed as follows:

(30) $$S_{T}=S_{\theta ,Total}+S_{\Psi ,Total}+S_{mf,Total}$$

The global entropy production due to heat transfer fluid friction in the magnetic field are represented by *S_θ,Total_*, *S_ψ,Total_*, and *S_mf_*_,*Total*_. These quantities are derived by the integration of the local entropy-generating components throughout the whole domain:

(31) $$S_{\theta ,Total}=\int_{\Omega }S_{\theta }\;d\Omega$$

(32) $$S_{\psi ,Total}=\int_{\Omega }S_{\psi }\;d\Omega$$

(33) $$S_{mf,Total}=\int_{\Omega }S_{mf}\;d\Omega .$$

The Bejan number is used to quantify the entropy generation:

(34) $$Be=\frac{S_{\theta }}{S_{\theta }+S_{v}+S_{mf}}.$$

*Be_avg_* is the average Bejan number and is computed as:

(35) $$Be_{avg}=\frac{S_{\theta ,Total}}{S_{\theta ,Total}+S_{\Psi ,Total}+S_{mf,Total}}.$$

## 4. Numerical Methodology, Validation, and Mesh Evaluation

**Figure 2:**
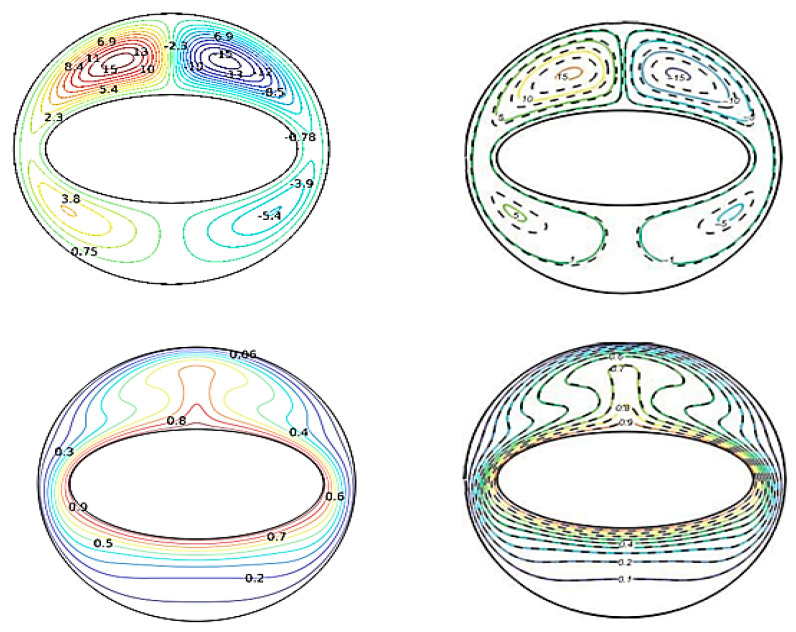
Comparison of streamlines and isotherm contours of the our results [**right**] and Sheikholeslami et al. [39] [**left**].

After discretization, the non-dimensional partial differential equations controlling transport equations as well as the associated boundary conditions are numerically solved by utilizing the finite element approach. The well-known Galerkin weighted residual approach is used to transform the governing equations from the partial differential from to a set of integral equations. The integration is carried out using Gauss’s quadrature. The numerical analysis method of Newton–Raphson is employed to address the integration equations. When defining the convergence criterion for the last iteration, it is important to note that the largest absolute relative error for all variables must be less than 10^−5^. Using the results presented by Sheikholeslami et al. [39], the validity of the current numerical approach has been verified by comparing the results of this study with those presented in that paper. Figure 2 depicts the flow behavior (streamlines) and the thermal behavior (isotherms).

The data given in Figure 2 for both flow and thermal characteristics exhibit a sufficient degree of precision. As a consequence, our numerical model can deliver dependable findings.

A thorough grid refinement research was conducted to ensure that the current investigation’s conclusions are independent of the grid sizes employed.

As demonstrated in Table 2, *Nu_avg_* and ${\left|\psi \right|}_{\max}$ values have been recorded for the various meshes. It is noted that the value of *Nu_avg_* and ${\left|\psi \right|}_{\max}$ changes by less than 0.1% when grid elements number is increased from 41,378 to 48,264. As a result, all simulations were conducted with a grid size of 41,378 elements.

## 5. Results and Discussion

This paper presents the flow behavior of free convection heat transmission in a copper water-based nanofluid in a porous annulus consisting of a cold wavy wall cylinder and a heated Koch snowflake-shaped wall cylinder. The streamlines (ψ), isotherms (*T*), and isotropic lines (*S*) are presented for different values of the Rayleigh number (*Ra*), Hartman number (*Ha*), Darcy number (*Da*), nanoparticle volume percentage (*φ*), and undulations number of the outer wavy cylinder (three cases).

**Figure 3:**
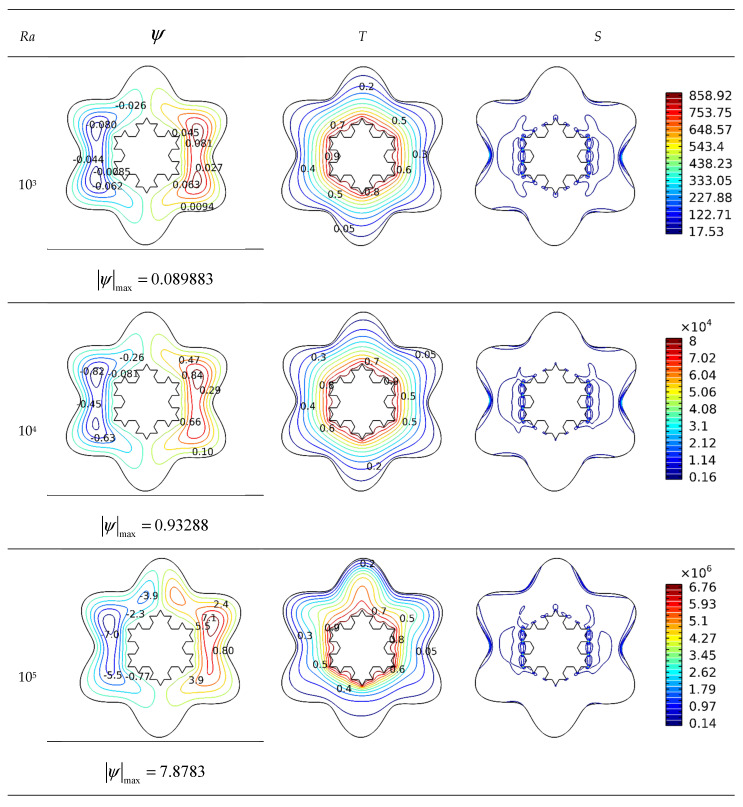

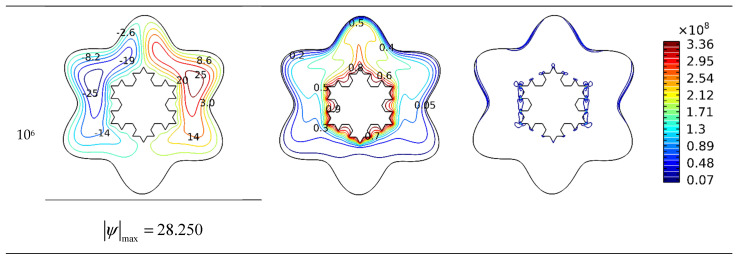
Ra number impact on streamlines and isotherms (*Da* = 10^−2^, *Ha* = 0, and *φ* = 0.04).

In Figure 3, the impact of raising the *Ra* number on the outlines of streamlines, isotherms, and isotropic lines for *Da* = 10^−2^, *Ha* = 0, and *φ* = 0.04 and *Ra* numbers 10^3^–10^6^ are displayed. The goal of defining these contours is to explore the effect of variations in buoyancy force in the enclosure on heat transport parameter performance. *Ra* may be increased by varying the temperature of the hot and cold sources. By raising this value, the buoyant force of the fluid is intensified, which is the primary source of circulatory movement. The increment of the flow intensity inside the cavity accelerates the flow, resulting in a more diverse temperature distribution between the hot and cold sources and the appearance of a plume in the isotherms. Zones with lower heat transmission will develop only in those sections of the cavity where the circulation restricted due to the unique construction of the flow barrier, where the temperature distribution is not uniform and has a greater value. Additionally, owing to the fluid’s indirect motion in these locations, flow dissipation is especially substantial at higher *Ra* values. As a result, the quantity of irreversibility in the vicinity of the hot boundary is considerable. Due to the augmentation of the flow velocity and the adherence of the flow to these surfaces, the quantity of entropy generated around the wavy cold wall is also significant.

**Figure 4:**
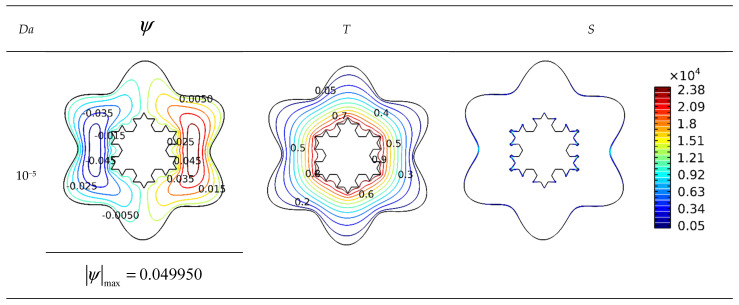

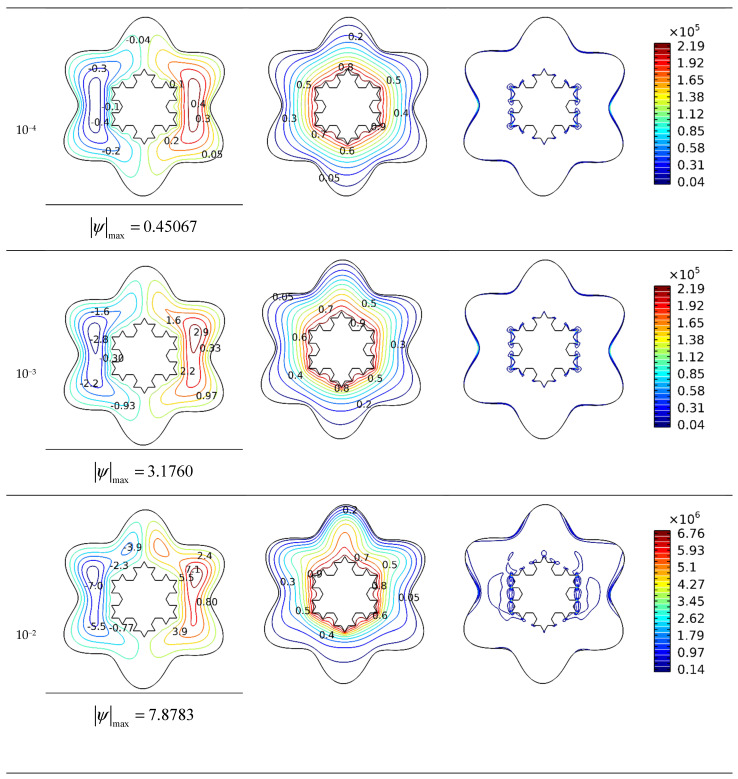
Influence of *Da* number on streamlines and isotherms (*Ra* = 10^5^, *Ha* = 0, and *φ* = 0.04).

In the contours of Figure 4, the streamlines, isotherms, and isotropic lines are represented as the Darcy number varies for *Ra* = 10^5^, *Ha* = 0, and *φ* = 0.04. In these profiles, the flow performance and heat transmission of the examined design are compared with the variations in the permeability of enclosure. As the *Da* number grows, the penetration of the flow cross-section in the enclosure improves, and the flow circulates in the enclosure with less dissipation owing to the buoyancy force. Therefore, for increasing *Da* numbers, the flow intensity is enhanced, and the mass flow rate is heightened at each segment of the cavity. On the other hand, thermal transfer augments with enhancing *Da* number, and the temperature gradients diminish. The produced irreversibility in the enclosure is impacted by the lowering of the *Da* number owing to the intensification of the hot patches and the decline of the flow rate in the lower *Da* numbers. By multiplying the *Da* number, heat will be spread more uniformly across the flow layers and in the hollow sections.

**Figure 5:**
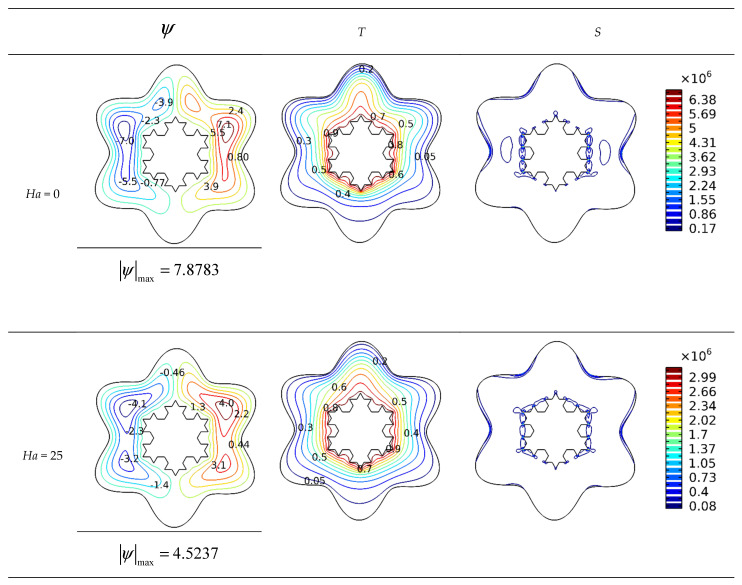

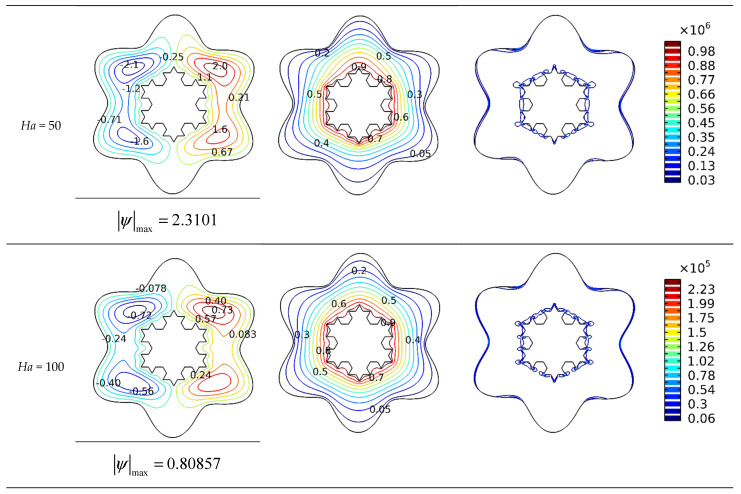
Influence of *Ha* number on streamlines and isotherms (*Ra* = 10^5^, *Da* = 10^−2^, and *φ* = 0.04).

The contours of Figure 5 demonstrate the impact of raising the *Ha* number on the streamlines, isotherms, and isotropic lines for the values of *Ra* = 10^5^, *Da* = 10^−2^, and *φ* = 0.04. These contours relate the flow performance and temperature distribution changes in the examined geometry to variations in the Lorentz force represented by Hartmann number. When applied in the opposite direction of the flow, Lorentz force depletes the flow velocity. Additionally, if the magnetic field is not aligned with the gravity pull in the inner cold portions, it will cause the fluid to move slowly away from the cold regions, which will create parallels isotherms, indicating that conductive heat transfer becomes dominant in the cavity when the Hartmann number rises. The nanofluid flow between the surfaces of the hot Koch snowflake and the surrounding area will move slowly due to the Lorentz force and even slower between the angled surfaces, thus increasing the cavity temperature in these zones, particularly those near the source. Due to the velocity degradation in the vicinity of the hot source, the quantity of entropy created has declined.

**Figure 6:**
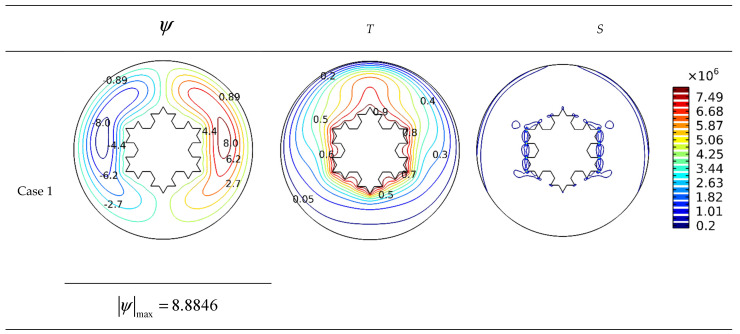

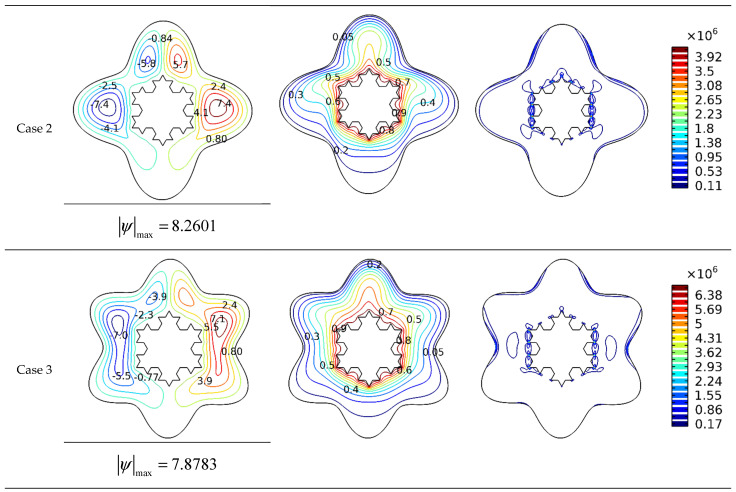
The influence of outer cylinder wall undulation number on streamlines and isotherms (*Ra* = 10^5^, *Da* = 10^−2^, *Ha* = 0, and *φ* = 0.04).

In the contours of Figure 6, the impact of undulation number of the outer cylinder on the streamlines, isotherms, and isotropic lines for the values of *Ra* = 10^5^, *Da* = 10^−2^, and *φ* = 0.04 in the state without applying a magnetic field is given. In these contours, the flow and heat transfer performance of the studied geometry is described and plotted with changes in the undulation number of the outer cylinder.

**Figure 7:**
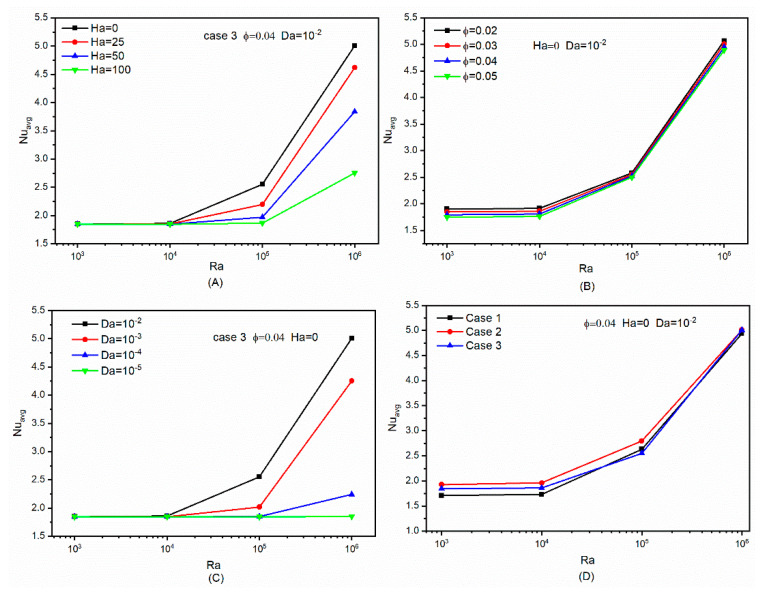
Impact of different factors on *Nu_avg_*: (**A**) impact of *Ha* number, (**B**) impact of volumetric fraction, (**C**) impact of *Da* number, and (**D**) impact of undulation number of outer cylinder wall.

The changes in the average Nusselt number as a function of factors such as the number of undulations, the *Ha* number, the *Da* number, and the volumetric fraction of nanoparticles are depicted in Figure 7 as a function of *Ra* numbers from 10^3^ to 10^6^. Figure 7A depicts the impact of different Hartmann numbers on the average Nusselt number as a function of the *Ra* number. The rise in *Ha* results in significant changes at higher values of *Ra* number in these graphs. Indeed, owing to the orientation of the magnetic field, the Lorentz force is hindering the buoyancy forces. Thus, increasing the *Ha* number hinders the convective flow of the nanofluid, which reduce the average Nusselt number. At *Ra* values less than 10^4^, the profile of Figure 7A indicates that free convection is weak and heat transmission is comparable to conduction, and raising the *Ha* value does not affect the rate of heat transfer. With a rising *Ra* number, the application of Lorentz force with varying intensities becomes critical owing to the necessity of flow circulation inside the cavity. Applying a Lorentz force with a larger *Ha* number inhibits heat transmission on hot and cold surfaces owing to junction balancing and flow separation. The average Nusselt number is presented for variable nanoparticles concentrations in Figure 7B. This research is conducted for the state *Da* = 10^−2^, without the application of a magnetic field. Due to their enhancing effect on the thermal conductivity, raising the inclusion of solid nanoadditives in the base fluid facilitates heat transmission. Increases in the aforesaid attribute may greatly enhance the Nusselt number in the examined geometry in particular areas of the cavity. On the flip side, by incorporating more solid nanoparticles into the base liquid, the viscosity may be increased. These rises in the viscosity of the nanofluid may reduce the intensity of the flow. Generally, raising the concentration of solid nanoparticles as shown in the plots of Figure 7B has had a modest effect on the increase of the Nusselt number, and this remain true for practically all *Ra* values. The impact of changing the *Da* number on the mean Nusselt number behavior is shown in the diagrams of Figure 7C. The porous media permeability inside the enclosure grows as the *Da* number increases. Reduced the porous medium permeability obstructs and depletes the nanofluid circulation motion. The thermosyphon force is the primary mechanism of fluid motion in the enclosure. This mechanism was formed as a result of the nanofluid density difference between the hot and cold zones of the cavity. By obstructing the flow and lowering the *Da* number, the heat transfer rate is weakened. The trend seen in Figure 7C has resulted in a decreased Nusselt number. While the *Ra* number increases when the permeability drops, the Nusselt number falls as the fluid motion elements weaken and depreciate. The variations in Nusselt number behavior caused by the number of undulations in the outer cylinder wall are depicted in the diagrams of Figure 7D for three distinct scenarios.

**Figure 8:**
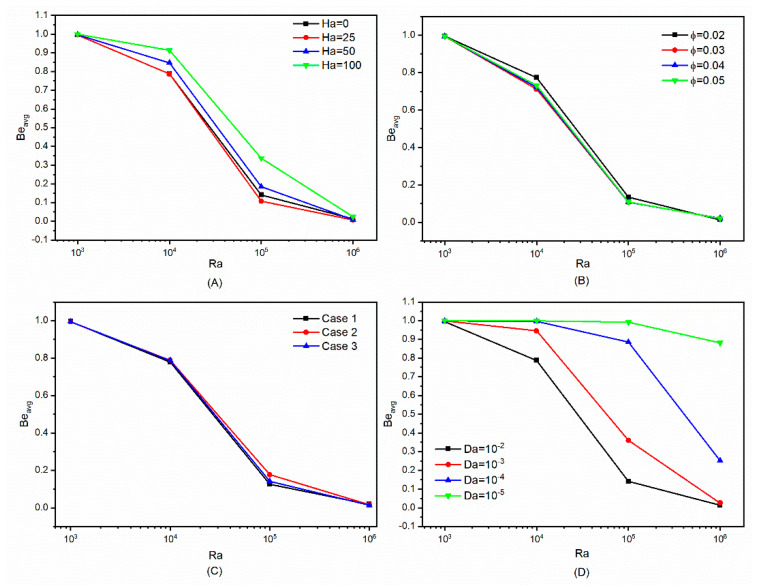
The impact of different factors on Bejan number: (**A**) impact of *Ha* number, (**B**) impact of volumetric fraction, (**C**) impact of undulation number of outer cylinder wall, and (**D**) impact of *Da* number.

The variations in the Bejan number are illustrated in Figure 8 for *Ra* values 10^3^ to 10^6^ as the *Ha* number, *Da* number, volumetric percentage of nanoparticles, and number of undulations vary. In Figure 8A, the values of the *Be* associated with various *Ha* and *Ra* numbers are displayed. Accelerating heat transfer between hot and cold sources is accomplished by increasing the *Ra* number and amplifying the flow rate inside the chamber. By increasing the *Ra* value, the irreversibility associated with insufficient heat dissipation and the formation of hot zones is reduced, and the behavior of Bejan number diagrams trends toward zero. By increasing the *Ra* value and optimizing fluid motion and heat dispersion, the friction factor may be increased. Lorentz force variations have resulted in substantial changes in the Bejan number in these graphs. The range of changes in these graphs demonstrates that for low *Ra* values, the Bejan number tends to be 1, suggesting a considerable rise in cavity temperature gradients and growth of the thermal boundary layer at low nanofluid velocities. By increasing the *Ha* number, the irreversibility is strengthened and the Bejan number values are strengthened. When *Ra* values are low, raising the *Ha* value increases fluid stagnation and therefore the temperature differential. At higher *Ra* values, the presence of high-intensity Lorentz forces impairs fluid flow and raises the friction factor. Due to the low velocity of the fluid at *Ra* number 10^3^, raising the *Ra* number has an influence on the Bejan number changes, and its value is determined only by the expansion of temperature gradients and is nearly constant. Due to the intensification of the fluid motion caused by the increase in buoyancy force, the Bejan number is not dependent on the Lorentz force or its intensity in *Ra* number 10^6^, and the Bejan number is a constant value.

In the diagrams of Figure 8B, the behavior of Bejan number values is displayed as a function of the volumetric percentage of nanoparticles added. Increases in the volumetric fraction of nanoparticles have a limited influence on changes in the Bejan number. The inclusion of nanoparticles may cancel out fluctuations in the Bejan number at various volume fractions, and the graphs show a nearly steady trend. Increased viscosity and density may be attained when adding more solid nanoparticles to promote heat transmission in the nanofluid. Furthermore, viscosity alters the friction factor’s behavior and increases the irreversibility of friction. Improved thermal conductivity, on the other hand, decreases temperature gradients and mitigates irreversibility caused by a non-uniform temperature distribution.

The illustrations in Figure 7C show the effects of modifying the undulations number of outer cylinders in the chamber on the Bejan number for three distinct scenarios. Limited variations in the Bejan number may be caused by altering the number of undulations in the cavity owing to interruption of the normal circulation of the fluid.

The curves in Figure 8D illustrate the trends of Bejan number behavior as a function of changes in *Da* and *Ra* numbers. The presence of a porous media in a cavity with varying degrees of permeability may have a considerable effect on the movement of the fluid, resulting in dramatic fluctuations in the flow velocity degradation. The reduction in *Da* number in the flow cross-sectional area impedes fluid’s movement. Permeability diminishes as the *Da* number falls and the temperature gradient grows. In summary, changes in cavity location, volume fraction, and *Da* number decrease relative to *Ha* number increase may all accelerate the formation of temperature gradients and the associated irreversibility.

## 6. Conclusions

The current study investigated the natural and laminar motion of a copper/water nanofluid with a volumetric fraction of between 2 and 5% inside a porous annulus formed by a Koch snowflake and a wavy cylinder. The primary interest of this numerical work is exploring the impact of different factors, including variations in the number of undulations in the outer wavy cylinder, application of magnetic force, and a rise in *Ra* and *Da* numbers on the natural convection of a porous annulus cavity. The changes in isotherms, streamlines, isotropic lines, and average Nusselt and Bejan numbers for the aforementioned physical parameters were investigated.

The following points summarizes the research’s major findings:

- Creating a higher intensity flow in the annulus by raising the Rayleigh number results in boosted free convective flow in the enclosure, which accelerates the heat transfer and the irreversibility due to fluid friction.
- Due to the unique shape of the heated Koch snowflake, nanofluid becomes trapped between the angled portions, causing the temperature distribution in these locations to be irregular and have a higher value.
- The cavity’s produced irreversibility is influenced by the decline in *Da* number, lower *Da* numbers cause the intensification of temperature in hot regions and a decrease in fluid velocity. By increasing the *Da* value, heat is transferred more equally across the flow layers and increases the entropy production due to fluid friction.
- At the highest studied *Re* number, decreasing the *Da* number from 10^−2^ to 10^−5^ resulted in magnifying *Be_avg_* by 18 times.
- Due to the direction of the magnetic field, applying Lorentz’s force decreases the average Nusselt number; however, this impact was less noticeable for lower Rayleigh numbers than at higher ones.
- At the highest studied *Re* number, *Nu_avg_* was enhanced by 100% when the *Ha* number was decreased from 100 to 0, while it was increased by 200% when the *Da* number increased from 10^−5^ to 10^−2^.

## Figures and Tables

**Table 1:** Thermophysical characteristics of (Cu/water) [35].

Properties	ρ	*Cp* (J/kg K)	*k* (W/m K)	*σ* (S/m)	*β* (K^−1^)
H_2_O (base fluid)	997.1	4179	0.613	5.5 × 10^−6^	21 × 10^−5^
Cu (nano-particle)	8933	385	400	59.7 × 10^6^	1.67 × 10^−5^

**Table 2:** *Nu_avg_* and ${\left|\psi \right|}_{\max}$ for various grid resolutions.

Grid Resolutions	4546	6894	17,038	41,378	48,264
*Nu_avg_*	2.1625	2.2460	2.4543	2.5466	2.5503
${\left|\psi \right|}_{\max}$	7.8165	7.8293	7.8612	7.8768	7.8783

## Abbreviations

**List of symbols**	
*Cp*	Heat capacitance (J kg^−1^ K^−1^)
*g*	Gravity (m s^−2^)
*Ha*	Hartmann number
*Nu*	Nusselt number
*p*	Pressure (Nm^−2^)
*P*	Dimensionless pressure
*Pr*	Prandtl number
*R*	Base circular radius of the block (m)
*Ra*	Rayleigh number
*T*	Dimension temperature (K)
*u*, *v*	Dimensional velocity components along x and y directions (m s^−1^)
*U*, *V*	Non-dimensional velocity components along with x and y directions
*x*, *y*	Cartesian coordinates (m)
*X*, *Y*	Non-dimensional coordinates
**Greek symbols**	
*A*	Thermal diffusivity (m^2^ s^−1^)
*β*	Thermal expansion coefficient (K^−1^)
*k*	Thermal conductivity (W m^−1^K^−1^)
*μ*	Dynamic viscosity, kg m^−1^ s
*υ*	Kinematic viscosity (m^2^ s^−1^)
*ρ*	Density (kg m^−3^)
*ϕ*	Volume fraction of the nanoparticles
*θ*	Non-dimensional temperature
**Subscripts**	
*C*	Cold	
*h*	Hot	
*F*	Fluid	
*P*	Solid particles	
*S*	Solid block	
*Avg*	Average	
